# *Lactobacillus Paracasei CNCM I 1572* is Better than Placebo in Preventing Acute Diverticulitis Occurrence (Revised Manuscript 661a120f-b910-4133-ab7e-4bd3e1713c96)

**DOI:** 10.1007/s12602-025-10812-y

**Published:** 2025-10-17

**Authors:** Antonio Tursi, Marcello Picchio, Walter Elisei, Giovanni Brandimarte, Francesco Di Mario, Silvio Danese, Alfredo Papa

**Affiliations:** 1 Territorial Gastroenterology Service, ASL BAT, Andria, BT Italy; 2 Department of Medical and Surgical Sciences, School of Medicine, Catholic University, Rome, Italy; 3 Division of Surgery, “P. Colombo” Hospital, ASL RMH, Velletri, Rome, Italy; 4 Division of Gastroenterology, “S. Camillo” Hospital, Rome, Italy; 5 Humanitas University Consortium, Rome, Italy; 6 University of Parma, Parma, Italy; 7 Gastroenterology and Endoscopy, IRCCS Ospedale “San Raffaele” and University “Vita-Salute, San Raffaele, Milan, Italy; 8 Department of Translational Medicine and Surgery, Università Cattolica del S. Cuore, Rome, Italy; 9 Digestive Diseases Centre (CEMAD), Department of Medical and Surgical Sciences, Fondazione Policlinico Universitario “A. Gemelli”, IRCCS, Largo Francesco Vito, 1, 00168 Rome, Italy

**Keywords:** Acute diverticulitis, Controlled study, Placebo, Probiotics, Symptomatic uncomplicated diverticular disease

## Abstract

Data analysing the impact of probiotics in preventing the occurrence of acute diverticulitis (AD) in patients having symptomatic uncomplicated diverticular disease (SUDD) of the colon are still scarce. We assessed the effectiveness of *Lactobacillus paracasei* CNCM I 1572 in preventing the first episode of AD in patients with SUDD. A post-hoc analysis of a 12-month, multicentre, double-blind, placebo-controlled study was conducted. One hundred and five patients were randomly enrolled in a double-blind fashion into Group L (*Lactobacillus paracasei* *CNCM I 1572*, 24 billion CFU/day) and Group P (placebo). Patients received treatment for 10 days per month for 12 months. The occurrence of AD was defined according to radiological criteria from abdominal computerised tomography. AD occurred in 7 of the analysed patients during follow-up: 6 patients were in group P and one patient in group L (*p* = 0.036). AD was uncomplicated in 6 out of 7 patients, while one patient receiving placebo underwent surgery due to free perforation. During the follow-up, mild adverse events (7.27% in group L and 9.0% in group P, *p* = 0.90) were recorded and did not necessitate treatment discontinuation. *Lactobacillus paracasei* CNCM I 1572 appears to be more effective than placebo in preventing the first episode of AD in SUDD patients. It could therefore be a real chance for the prevention of this diverticular disease complication. We need more data from prospective placebo-controlled trials enrolling a large population of patients to confirm this finding.

**Supplementary Information:**

The online version contains supplementary material available at 10.1007/s12602-025-10812-y.

## Introduction

The diverticulosis of the colon is an anatomical alteration characterised by the presence of outpouchings through the colon [1]. The prevalence is higher in the Western world, but it is rapidly increasing in both the Eastern and the developing world [1].

Diverticulosis remains asymptomatic throughout life in most people, and only one-fifth of them develop clinical manifestations linked to diverticula, the so-called diverticular disease (DD) [1]. Like diverticulosis, DD has also shown a significant increase worldwide [1].

Symptomatic Uncomplicated Diverticular Disease (SUDD) is the primary form of DD, affecting approximately 80% of patients with DD. In contrast, the prevalence of acute diverticulitis (AD), the most severe form of DD, is estimated to be around 20% of patients with DD [1–4]. SUDD is characterised by left-lower, long-standing (≥ 24 h) quadrant pain [5, 6], criteria that can be successfully applied to both Western and Eastern populations [7].

SUDD is characterised by impaired quality of life, with a double incidence of acute diverticulitis (AD) than patients with asymptomatic diverticulosis, incidence estimated as about 10% over 10 years [8]. Regarding the pathophysiology of DD, new interesting data have confirmed the role of gut microbiota (GM) perturbation in the occurrence of SUDD and AD [9], opening the door to GM modulation as a therapeutic option in managing these patients. Currently, no therapy is advised to prevent recurrence of AD [10, 11], and few data are available about the role of drugs in preventing the first occurrence of the disease [10, 12]. Since a previous controlled study found that mesalazine, with or without probiotics, was more effective than placebo in controlling SUDD symptoms [13], we performed a post-hoc analysis of that trial to assess whether *Lactobacillus paracasei* CNCM I 1572 was more effective than placebo in preventing the occurrence of AD.

## Patients and Methods

### Setting and Participants

This is a post-hoc analysis of a multicenter, randomised, double-blind, double-dummy, parallel-group, placebo-controlled trial previously published [13]. The trial enrolled 250 consecutive outpatients (aged > 18 years) with SUDD who had achieved clinical remission and were treated with mesalazine, with or without probiotics, probiotics only, or placebo, to maintain remission through a one-year follow-up.

In detail, patients were enrolled after experiencing a previous episode of SUDD in the absence of a prior history of AD. They remained asymptomatic following a 4-week course of mesalazine 2.4 g/day or a 10-day course of non-absorbable antibiotics (rifaximin, 800 mg/day) or a 2-week course of probiotics (Lactobacillus paracasei CNCM I 1572, Lactobacillus casei DG, as previously defined in the protocol, 24 billion/day). SUDD was defined as the presence of abdominal pain recorded in the lower left quadrant, lasting for > 24 consecutive hours [5, 6]. Although the definition of SUDD is still tricky, abdominal pain is considered the most critical symptom in SUDD. Its behaviour is regarded as the best tool to differentiate between SUDD and Irritable Bowel Syndrome (IBS): abdominal pain in SUDD generally exhibits a long-lasting behaviour, whereas abdominal pain in irritable bowel syndrome typically exhibits a short-lasting behaviour [8, 9]. Abdominal pain was assessed using a 10-point visual scale (0: absence of pain, 10: severe pain).

Exclusion criteria of the trial were: IBS by the ROME III criteria [5, 6]; celiac disease by anti-endomysium and anti-transglutaminase antibodies assessment; thyroid diseases by thyroid-stimulating hormone, free-thyroid hormone 3 and 4; bacterial and/or parasitic intestinal diseases by stool cultures; current or previous history of AD, diagnosed by abdominal computerized tomography or by ultrasounds, and defined as inflammation of colonic wall harboring diverticula with fat stranding, and with or without complications such as abscesses, stenosis or fistulas (namely uncomplicated or complicated diverticulitis) [1].

The presence of colonic diverticula was evaluated by colonoscopy. The extent of diverticulosis was assessed by subdividing the colon into four segments (ascending colon, transverse colon, descending colon, and sigmoid colon) and graded according to the number of segments involved, ranging from 1 (one segment involved) to 4 (entire colon). The severity of diverticulosis was evaluated using the following arbitrary scale: mild (< 5 diverticula per segment), moderate (5–10 diverticula per segment), and severe (> 10 diverticula per segment).

The Ethics Committee of each recruiting centre approved the protocol. The study was conducted in accordance with Good Clinical Practice (GCP) and with the Declaration of Helsinki. All patients gave written informed consent for their participation. All authors had access to the study data and had reviewed and approved the final manuscript.

The trial was registered with www.ClinicalTrials.gov, number NCT01534754. All procedures for the enrolment and conduct of the study (complete inclusion and exclusion criteria, study procedures, concomitant treatments, randomisation, assessment of compliance, etc.) have been previously published with the trial and are now available as supplementary methods.

### Treatment

In this post-hoc analysis, we analysed the patients who were randomly assigned to one of the following treatment groups:

- Group L. Active *Lactobacillus paracasei CNCM I 1572*, 1 sachet/day with 24 billion bacteria for 10 days/month.
- Group P. *Lactobacillus paracasei CNCM I 1572* placebo, one sachet/day for 10 days/month.

The placebo was in the form of sachets identical to those containing active Lactobacilli. *Lactobacillus paracasei CNCM I 1572* (Enterolactis Plus®), as well as the placebo, were supplied by the manufacturing company (Sofar S.p.A., Trezzano Rosa (MI) – Italy, now a branch of AlfaSigma S.p.A., Bologna—Italy) for the entire duration of the trial. The supply of the experimental drugs was the only involvement of Sofar S.p.A. in this study; there was no involvement, nor funding, from AlfaSigma S.p. A. A was obtained for this post-hoc analysis.

### Primary Endpoint

The primary endpoint was the rate of patients developing AD during a 12-month follow-up. Diagnosis of AD was made according to the above-reported radiological criteria, and abdominal computerised tomography was performed in every case of suspected acute diverticulitis symptoms (e.g., the occurrence of abdominal pain associated with fever).

### Secondary Endpoint

As a secondary point, we assessed the safety of the drug, as adverse events linked to the product are recorded through the follow-up.

### Statistical Methods

Categorical variables were expressed as absolute values and percentages in the text and tables, while continuous variables were expressed as medians and interquartile ranges (IQRs). Statistical analysis was performed using the chi-square test for categorical data and the Mann–Whitney test for continuous data. We analysed data using an intention-to-treat analysis to estimate the probability of AD occurrence and the persistence of clinical remission, as determined using the Kaplan–Meier method. Differences between curves were evaluated using the log-rank test. The relative risk (RR) and 95% CI were calculated for the two study groups. A two-tailed *P* value of 0.05 was considered statistically significant. The collection and analysis of data were performed using MedCalc Release 14.8.1

## Results

**Fig. 1:**
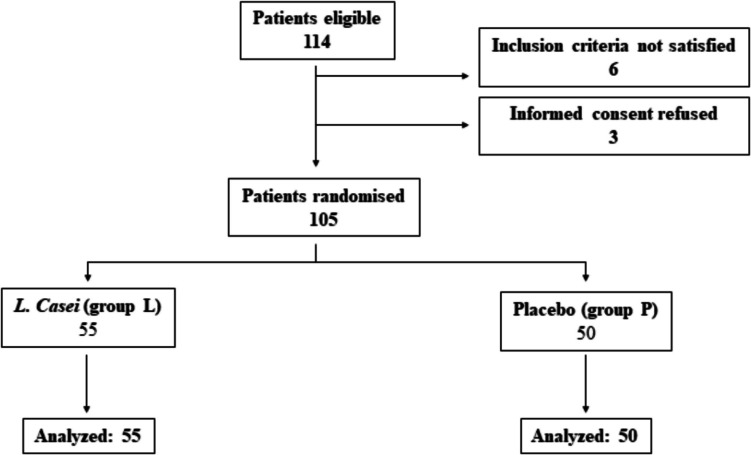
Flow-chart of the study. Group L: active Lactobacillus casei; group P: placebo

All 105 patients enrolled in the study completed it: 55 were treated with Lactobacillus paracasei CNCM I 1572 (Group L) and 50 received a placebo (Group P). The flow-chart of the study is reported in Fig. 1.

Patient demographics and clinical characteristics are reported in Table 1.

**Table 1:** Baseline demographic and clinical characteristics by treatment group

	**Group L (55 pts)**	**Group P (50 pts)**	P*
Age, years	64 (61–68)	62 (58–70)	0.416
Sex male	27 (49.1)	30 (60.0)	0.265
First diagnosis of SUDD	26 (47.2)	25 (50.0)	0.762
Symptom duration before diagnosis	1 (1–12)	1 (1–24)	0.377
Presence of co-morbidities	37 (67)	36 (72.0)	0.601
Charlson’s score = 1	12 (21.8)	12 (24.0)	0.790
Concomitant ASA	7 (12.7)	9 (18.0)	0.452
Concomitant calcium channel blockers	4 (7.3)	5 (10.0)	0.623
Concomitant anti-diabetic therapy	6 (10.9)	7 (14.0)	0.632
Therapy to obtain remission			
Antibiotics	23 (41.8)	22 (44.0)	0.821
Mesalazine	5 (9.0)	4 (8.0)	0.855
Probiotics	12 (21.8)	8 (16.0)	0.452
Extension			
One segment	40 (72.7)	37 (74.0)	
Two segments	9 (16.4)	6 (12.0)	0.743
Three segments	2 (3.6)	3 (6.0)	
Four segments	4 (7.2)	4 (8.0)	
Severity before obtaining remission			
Mild	2 (3.7)	3 (6.0)	
Moderate	26 (47.2)	26 (52.0)	0.653
Severe	27 (49.1)	21 (42.0)	
Abdominal pain	2 (0–4)	1 (0–4)	0.151

*Chi-square test for categorical data; Mann–Whitney test for continuous data

### Primary Endpoint

**Fig. 2:**
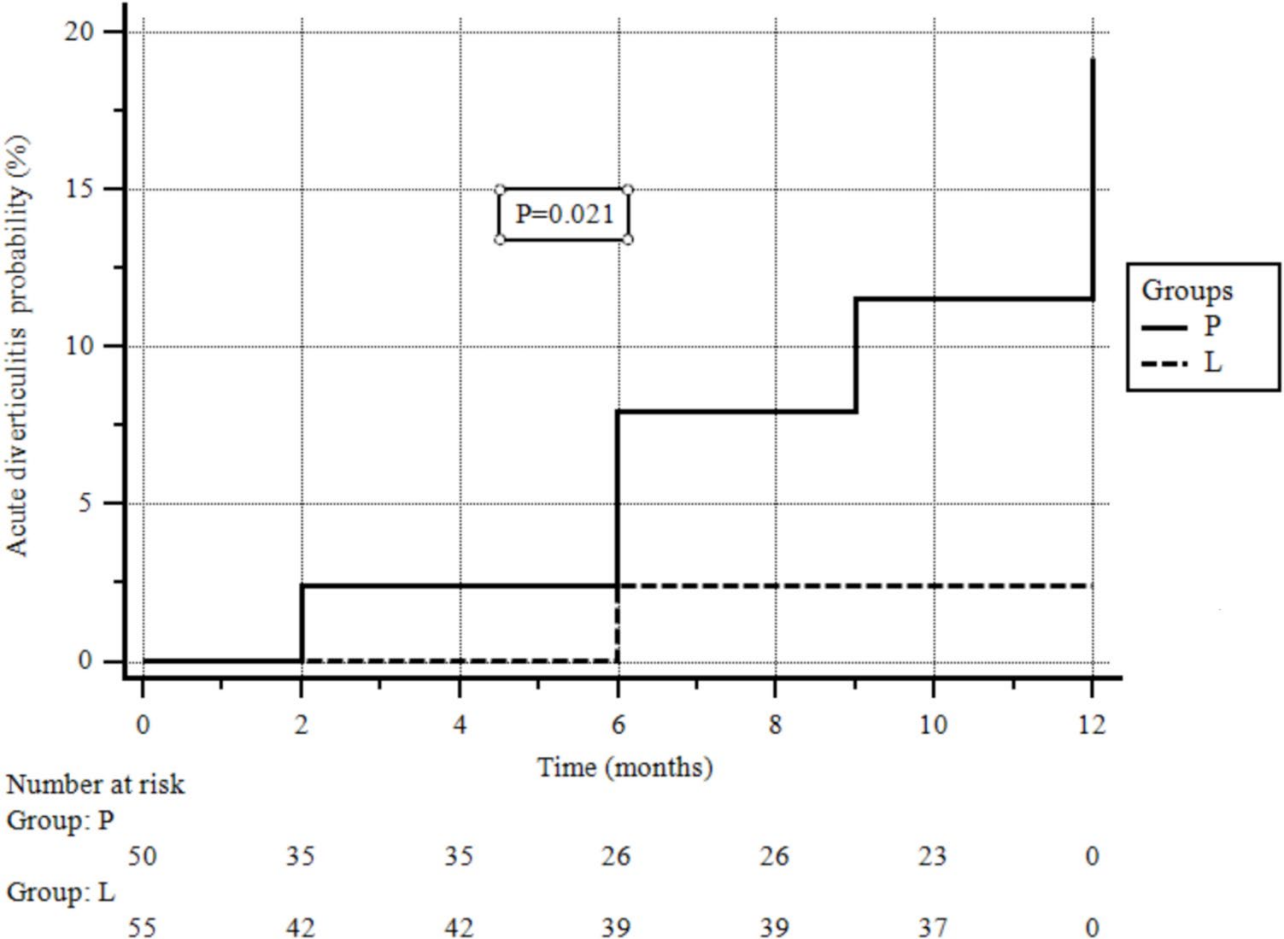
Kaplan–Meier analysis of cumulative rates of acute diverticulitis occurrence by study group. Group L: active Lactobacillus casei; group P: placebo. *p* = 0.021, log-rank test

AD occurred in 6/50 (12%) patients in group P versus 1/55 (1.8%) patients in group L (p = 0.036). Intention-to-treat and per-protocol analysis corresponded. AD was uncomplicated in 6 out of 7 patients, while one patient receiving placebo underwent surgery due to free perforation. In Fig. 2, the Kaplan–Meier analysis of cumulative rates of acute diverticulitis occurrence by study group is shown (p = 0.021). Univariate and multivariate analyses were not performed to assess the effect of baseline clinical characteristics on the occurrence of AD due to a lack of statistical power. However, 4 out of 6 patients developing AD had diverticulosis involving more than two segments and severe diverticulosis at baseline.

### Secondary End-Points

During the follow-up, 4 patients (7.3%: 1 case of flatulence, 2 case of bloating, and 1 case of belching were recorded) in group L, and 6 patients (9.0%: 2 cases of flatulence, 1 case of headache, 1 case of bloating, and 2 cases of diarrhea were recorded) in group *P* experienced adverse events during the entire follow-up (*p* = 0.90). All these symptoms were mild, and no patients stopped treatment, concluding the study.

## Discussion

The natural history of DD remains largely unknown, and the lack of robust, placebo-controlled studies has precluded any meaningful conclusions regarding the appropriate management of this disease.

This is particularly true for the prevention of AD occurrence. We know that AD may occur in up to 10% of SUDD cases [8, 14], a rate double that of simple diverticulosis [15, 16]; yet, several current practical guidelines do not provide any advice regarding treatment for the prevention of AD occurrence [10, 11].

GM perturbation is hypothesised to influence the aetiology of the disease and its complications [9]. It is hypothesised that microbial imbalance may be one of the key factors in the development of low-grade inflammation, which, together with visceral hypersensitivity and alterations in colonic motility, contributes to the occurrence of DD [1]. This hypothesis has been confirmed by several studies conducted in patients with AD [17–19] and patients with SUDD [20–24], which have found significant changes in pro-inflammatory taxa in these patients and suggest a relationship with the severity of the disease.

Based on this evidence, the restoration of GM for preventing diverticulitis occurrence using probiotics has been proposed for several years [25], and some placebo-controlled data are currently available. But while probiotics as an add-on treatment in AD have been successfully tested [26, 27], data on the prevention of AD are scarce. The only placebo-controlled study in this setting found that the probiotic mixture Symprove™ was better than placebo in preventing AD occurrence in SUDD patients treated for 90 days. Although the logistic regression indicated that this difference was not statistically significant (OR = 0.34; 95% CI 0.36–1.5; *P* = 0.19), the HR was 0.35 (95% CI 0.09–1.34), suggesting a tendency toward longer episode-free intervals in the probiotic-treated group [26]. It is clear that these results are influenced by the short-term duration of the study (90 days), and that a longer follow-up would be advisable to draw more robust conclusions.

This placebo-controlled study was conducted over a reasonable period (12 months) and yielded interesting findings.

First, in this study, we tested a single bacterial strain, the *Lactobacillus paracasei CNCM I 1572*. We demonstrated for the first time that this bacterial strain is significantly more effective than a placebo in preventing the occurrence of AD in SUDD patients. This point is of extreme interest to clinical practice because it provides clinicians with a therapeutic opportunity to prevent DD complications. This result is likely linked to the characteristics of this strain. It is a Gram-positive bacterial strain isolated from human faeces and usually present in healthy individuals’ intestinal microbiota, it has several interesting characteristics: produces lactic acid, providing a quick rebalancing action of GM [28]; does not induce antibiotics resistance, guaranteeing safe human consumption [29]; can stimulate the production of proinflammatory cytokines by antigen-presenting cells (APCs) responsible for the detection of microorganisms and involved in their clearance through phagocytosis [30]; acts as mild booster of the innate immunity, contributing to a more efficient and faster immune response against potential infectious agents [31]. In SUDD patients, it has been found to increase nitric oxide (NO)-mediated responses, with a decrease in inducible nitric oxide synthase (iNOS) expression and an increase in interleukin-10 release [32]. All their intrinsic characteristics, together with the restoration of GM imbalance, may therefore explain this critical result.

Second, all the clinical results were obtained using a cyclic course of probiotic treatment. Again, the characteristics of this strain seem to be the key to explaining these results. This strain appears to persist in the gastrointestinal tract for approximately two weeks after treatment discontinuation [33]. This is an important point because this persistence allows for cyclic administration with adequate patient compliance. Moreover, it seems to be effective against gram-negative anaerobes [34]. The results obtained confirm, therefore, that this bacterial strain may be particularly indicated in DD.

Of course, this study has some limitations.

The first one is that we analysed a selected population, namely patients with SUDD, who had never experienced AD in their history. We selected this population because SUDD patients may have an impaired quality of life with a risk of AD occurrence of about 10% [8]. This means that the results obtained cannot be generalised to all patients with diverticulosis, but only to those with SUDD. These results are therefore valid for the prevention of the first episode of AD only in SUDD patients without a previous history of AD.

The second one is that we don’t know the dietary habits of patients included in the study, which could influence the outcome of the disease. We know that patients with diverticulosis in the highest caloric quintiles and who consume mainly meat and ultra-processed foods are at higher risk of AD [35]. However, we don’t know whether this also occurs in patients with SUDD who have an intake of fibre, both soluble and insoluble, like that of healthy controls and asymptomatic diverticulosis [36]; however, it is unclear whether this may influence the onset of AD.

The third limitation is the absence of an objective evaluation of colonic inflammation, e.g., faecal calprotectin. This marker helps differentiate between functional and organic symptoms [37] and can aid in distinguishing between SUDD and IBS [6].

The fourth is that the impact of this bacterial strain on the GM was not assessed at baseline or during follow-up. This is likely because the original protocol was conceived more than ten years ago, when the GM assessment in real life was more complex to perform. Again, the old design of the study does not permit this post-hoc analysis to overcome this limit. Of course, this limitation is not a problem when considering the primary endpoint of this analysis, because all patients with moderate-to-severe abdominal pain underwent abdominal computerised tomography. Finally, the low number of events (namely, the low number of AD occurrences during the 12-month follow-up) did not permit the performance of a multivariate analysis that could identify risk and protective factors.

In conclusion, this post-hoc analysis found *that Lactobacillus paracasei CNCM I 1572* was more effective than the placebo in preventing the first episode of AD in SUDD patients. As discussed, this result has been reached thanks to the intrinsic characteristics of this bacterial strain. It could therefore be supposed to be a real therapeutic chance for clinicians to prevent the occurrence of AD.

However, more data from prospective studies, including patients' dietary habits and measurements of inflammatory markers, such as faecal calprotectin and GM analysis, are needed to provide further evidence for the use of this probiotic in preventing AD in patients with SUDD.

## Supplementary Information

Below is the link to the electronic supplementary material.

Supplementary file1 (DOC 57 KB)

## Data Availability

Data are available upon reasonable request.
