# Supplementary material for: Lactobacillus Paracasei CNCM I 1572 is Better than Placebo in Preventing Acute Diverticulitis Occurrence (Revised Manuscript 661a120f-b910-4133-ab7e-4bd3e1713c96)
Source: Probiotics Antimicrob Proteins. 2025 Oct 17;18(3):4738–45. doi: 10.1007/s12602-025-10812-y (PMC13176042; doi:10.1007/s12602-025-10812-y)
Supplement: Supplementary file 1 — Supplementary file1 (DOC 57 KB) [file 12602_2025_10812_MOESM1_ESM.doc]

**SUPPLEMENTARY METHODS OF THE MANUSCRIPT ENTITLED “*LACTOBACILLUS PARACASEI CNCM I 1572* IS BETTER THAN PLACEBO IN PREVENTING ACUTE DIVERTICULITIS IN PATIENTS WITH SYMPTOMATIC UNCOMPLICATED DIVERTICULAR DISEASE” (POST-HOC ANALYSIS OF THE TRIAL NCT01534754)**

***Setting and Participants***

We performed a post-hoc analysis of the population enrolled in the original multicentre, randomised, double-blind, double-dummy, parallel groups, placebo-controlled trial conducted from January 2009 to December 2010.

The original trial was conducted in order to investigate the most appropriate treatment for the prevention of SUDD relapse. Two hundred fifty consecutive outpatients (age >18 years) with SUDD were considered eligible for the study in the 14 participating centres. SUDD was defined as the presence of symptoms (mainly abdominal pain, but also constipation, diarrhea and bloating) in patients with diverticulosis, in the absence of any complication (stenoses, abscesses, fistulas),^1^ in whom the presence of abdominal pain was recorded in the lower left quadrant as lasting for >24 consecutive hours.^2,3^ Although the definition of SUDD is difficult and without standards, abdominal pain is considered the most important symptom in SUDD, and its behaviour is considered the best tool to differentiate between SUDD and Irritable Bowel Syndrome (IBS): abdominal pain in SUDD generally shows a long-lasting behaviour, whilst abdominal pain in IBS generally shows a short-lasting behaviour.^2,3^ Other competing diagnoses were excluded, in detail: IBS by the ROME III criteria; celiac disease by anti-endomysium and anti-transglutaminase antibodies assessment; thyroid diseases by thyroid-stimulating hormone, free-thyroid hormone 3 and 4; bacterial and/or parasitic intestinal diseases by stool cultures. Abdominal pain was assessed using a 10-point visual scale, assigning numerical values of 0 for absence of pain, 1-4 for mild pain, 5-7 for moderate pain and 8-10 for severe pain. SUDD was defined as the presence of an abdominal pain score ≥5 for at least 24 consecutive hours in patients with diverticulosis, in the absence of any complication (stenoses, abscesses, fistulas). The same scale was used to evaluate other symptoms assessed (diarrhea, constipation, rectal bleeding, bloating, sensation of incomplete evacuation, and mucorrhea). At enrolment, all patients were asymptomatic.

The presence of colonic diverticula was evaluated by colonoscopy. The extent of diverticulosis was assessed by subdividing the colon into four segments (ascending colon, transverse colon, descending colon, and sigmoid colon) and graded according to the number of segments involved, ranging from 1 (one segment involved) to 4 (entire colon). The severity of diverticulosis was evaluated using the following arbitrary scale: mild (< 5 diverticula per segment), moderate (5-10 diverticula per segment), and severe (> 10 diverticula per segment).

The original study was conducted in three university hospitals, ten general hospitals, and one ambulatory service. The Ethics Committee of each recruiting centre approved the protocol. The study was conducted in accordance with the principles of Good Clinical Practice (GCP) and the Declaration of Helsinki. All patients gave written informed consent for their participation. All authors had access to the study data and had reviewed and approved the final manuscript. This trial was registered with www.ClinicalTrials.gov, number NCT01534754.

## Sample Size

In the original trial, a minimum of 50 patients was required for each group. This calculation was based on the assumption that a continuity-corrected chi-square test with a type I error of 0.01 and a type II error of 0.05 for multiple comparisons was expected to detect a difference between the placebo group's success rate of 48% and the mesalamine group's success rate of 85%.^5^

## Study procedures

The study procedures were conducted for each patient enrolled in the study. At the screening visit, demographic characteristics, medical history, and current medications were recorded for each patient. A pregnancy test was also performed in women of childbearing age to exclude pregnancy.

All adverse events, as well as symptoms, were documented, classified, and graded. Study participants were provided with diary cards to assess and record their symptoms at entry and during follow-up. The patients were followed during the study and reassessed at 1, 2, 6, 9 and 12 months after starting treatment. The study duration was 12 months.

All study medications, including all empty packages and empty blisters, were required to be returned to the Centre to allow a drug accountability check before all residual medication was destroyed. In accordance with GCP guidelines for clinical studies, any surplus medication and all packages were returned to the Centre even in the case of withdrawal or discontinuation of the study.

## Concomitant treatments

Concomitant medications were permitted during the study course, provided they were used at a constant dosage and had been initiated at least one month before the baseline visit. All concomitant medications were recorded in the case report form (CRF), including the dosage and either the trade or generic name. The use of laxatives was permitted only if absolutely necessary, but lactulose was not allowed during the study period. The investigator was authorised to administer any supportive therapy considered essential for the patient’s health. These concomitant therapies were also recorded on the CRF. A regular diet, avoiding a high-fibre diet during the study, was advised, since a high-fibre diet may be a confounding factor for response to treatment.^8^

## Randomisation

Eligible patients were randomly assigned to receive active drugs or placebo using a computer-generated randomisation list.

Randomisation was carried out in a double-blind manner in blocks of two subjects using 1:1 allocation to the investigation groups.

## Assessment of compliance

The number of medication boxes dispensed and returned throughout the study was used to measure patient compliance (at least 80% of the prescribed drugs must be taken). This analysis was conducted descriptively to compare the treatment groups during the study.

## Inclusion criteria

Subjects were required to meet all the following inclusion criteria to be eligible for participation:

- males and females aged >18 years;
- diverticulosis showed by colonoscopy no more than 6 months prior to study entry;
- symptomatic episode of symptomatic uncomplicated diverticular disease achieving successful remission, occurring no more than 4 weeks before the study entry;
- patients who have given their free and informed consent.

A negative pregnancy test at the screening visit, agreement to use a valid contraceptive method for the duration of the study, patients not requiring hospitalisation, and patients willing and able to provide written informed consent were also considered inclusion criteria.

## Exclusion criteria

Subjects who met any of the following exclusion criteria were not enrolled in this study:

- current or previous diagnosis of acute diverticulitis (both complicated and uncomplicated);
- diverticular colitis;
- active or recent peptic ulcer;
- chronic renal insufficiency;
- allergy to salicylates;
- patients with intended or ascertained pregnancy, lactation;
- women of childbearing age not using contraceptives;
- lactulose-lactitol use in the two weeks before the enrolment and during the study;
- presence of diverticulitis complications (fistulas, abscesses, and/or stenoses);
- use of probiotic preparations, either prescribed or over-the-counter, within two weeks before the study entry;
- inability to give a valid informed consent or to properly follow the protocol;
- patients with active malignancy of any type, or history of a malignancy (patients with a history of other malignancies that have been surgically removed and who have no evidence of recurrence for at least five years before study enrolment were also acceptable);
- recent history or suspicion of alcohol abuse or drug addiction;
- any severe pathology that can interfere with the treatment or the clinical or instrumental tests of the trial;
- use of nonsteroidal anti-inflammatory drugs for one week before and throughout the study period (only acetaminophen was permitted).

Significant hepatic, renal, endocrine, respiratory, neurological or cardiovascular diseases, as determined by the investigator, were also considered exclusion criteria. Other exclusion criteria were as follows: history of severe adverse reactions or known hypersensitivity to maltose and/or silicon dioxide; patients requiring hospitalisation; use of any investigational drug and/or participation in any clinical trial within 3 months before this study; inability to give valid written informed consent or to properly follow the protocol.

## Treatment

The analyzed patients were randomly assigned to one of the following treatment groups:

- Group L. Active *Lactobacillus paracasei CNCMI 1572*, 1 sachet/day for 10 days/month;
- Group P. *Lactobacillus paracasei CNCMI 1572* placebo, 1 sachet/day for 10 days/month.

*Lactobacillus paracasei CNCMI 1572* (Enterolactis Plus®) formulation comprises sachets, each containing 24 billion viable lyophilized bacteria containing *Lactobacillus paracasei CNCMI* 1572 (L*actobacillus casei subsp. DG*)*,* administered as a single sachet per day for 10 days/month. The placebo consisted of sachets identical to those containing active Lactobacilli.

## Primary endpoint

The primary endpoint was the rate of SUDD patients developing AD during a 12-month follow-up after achieving remission of the SUDD. Diagnosis of AD was made according to the above-reported radiological criteria, and abdominal computerised tomography was performed in every case of suspected acute diverticulitis symptoms (e.g., the occurrence of abdominal pain associated with fever).

In detail, patients were enrolled after experiencing a previous episode of SUDD in the absence of a prior history of AD. They remained asymptomatic following a 4-week course of mesalazine 2.4 grams/day or a 10-day course of non-absorbable antibiotics (rifaximin, 800 mg/day) or a 2-week course of probiotics (Lactobacillus paracasei CNCM I 1572, Lactobacillus casei subsp. DG as previously defined in the original protocol, 24 billion/day).

## Secondary end-points

As a secondary point, we assessed the safety of the drug, as adverse events linked to the product are recorded through the follow-up.

## Statistical methods

Categorical variables were expressed as absolute values and percentages in the text and tables, while continuous variables were expressed as medians and interquartile ranges (IQRs). Statistical analysis was performed using the chi-square test for categorical data and the Mann-Whitney test for continuous data. We analysed data using an intention-to-treat analysis, estimating the probability of AD occurrence and persistence of clinical remission using the Kaplan-Meier method. Differences between curves were evaluated using the log-rank test. The relative risk (RR) and 95% CI were calculated for the two study groups. A two-tailed P value of 0.05 was considered statistically significant. The collection and analysis of data were performed using MedCalc Release 14.8.1. W.E. and M.P. performed the analysis.

# REFERENCES

1) Tursi A, Scarpignato C, Strate LL, et al. [Colonic diverticular disease.](https://pubmed.ncbi.nlm.nih.gov/32218442/) Nat Rev Dis Primers 2020;6: 20

2) Cuomo R, Barbara G, Andreozzi P, et al.[Symptom patterns can distinguish diverticular disease from irritable bowel syndrome.](https://pubmed.ncbi.nlm.nih.gov/23992370/) Eur J Clin Invest 2013;43: 1147-1155

3) Tursi A, Elisei W, Picchio M, Giorgetti GM, Brandimarte G. [Moderate to severe and prolonged left lower-abdominal pain is the best symptom characterizing symptomatic uncomplicated diverticular disease of the colon: a comparison with fecal calprotectin in clinical setting.](https://pubmed.ncbi.nlm.nih.gov/24583746/) J Clin Gastroenterol 2015;49: 218-221

4) [Papi C](http://www.ncbi.nlm.nih.gov/pubmed?term=Papi C%5BAuthor%5D&cauthor=true&cauthor_uid=7766741), [Ciaco A](http://www.ncbi.nlm.nih.gov/pubmed?term=Ciaco A%5BAuthor%5D&cauthor=true&cauthor_uid=7766741), [Koch M](http://www.ncbi.nlm.nih.gov/pubmed?term=Koch M%5BAuthor%5D&cauthor=true&cauthor_uid=7766741) et al. Efficacy of rifaximin in the treatment of symptomatic diverticular disease of the colon. A multicentre double-blind placebo-controlled trial. [Aliment Pharmacol Ther](http://www.ncbi.nlm.nih.gov/) 1995;9: 33-39

5) Tursi A, Brandimarte G, Giorgetti GM et al. [Mesalazine and/or Lactobacillus casei in preventing recurrence of symptomatic uncomplicated diverticular disease of the colon: a prospective, randomized, open-label study.](http://www.ncbi.nlm.nih.gov/pubmed/16633103) J Clin Gastroenterol 2006;40:312-316
